# Leg occlusion of the Ovation Alto stent graft main body caused by twisting of the stent graft: A case report

**DOI:** 10.1016/j.jvscit.2024.101625

**Published:** 2024-09-17

**Authors:** Masaaki Naganuma, Yukihiro Hayatsu, Ryoichi Tsuruhara, Hayate Nomura, Naoya Terao, Kazuhiro Yamaya, Masaki Hata

**Affiliations:** Department of Cardiovascular Surgery, Sendai Kosei Hospital, Sendai, Miyagi, Japan

**Keywords:** Abdominal aortic aneurysm, Endoleak, Endovascular abdominal aortic aneurysm repair, Occlusion, Ovation Alto stent graft

## Abstract

The Ovation Alto stent graft is a unique endovascular abdominal aortic aneurysm repair device. A low-viscosity, radiopaque liquid polymer fills the rings located within the legs of the device. We reported a rare case of a patient diagnosed with an abdominal aortic aneurysm who was surgically treated using endovascular abdominal aortic aneurysm repair with an Ovation Alto stent graft. Polymer underfilling due to contralateral leg twisting of the stent graft’s main body resulted in limb occlusion. We highlighted the importance of avoiding the rotation of the Ovation Alto stent graft, thus preventing the potential twisting of this device within the sheath after the insertion of the stent graft’s main body.

The Ovation Alto stent graft (Endologix Inc) is an endovascular abdominal aortic aneurysm repair (EVAR) device that has been commercially available since August 2020 and was developed based on the previous Ovation platform (Endologix Inc). It comprises a low-permeability polytetrafluoroethylene material. The proximal bare stent possesses a radial force, whereas the stent graft does not. Neck sealing is completed after polymer filling and ballooning by an integrated balloon. The graft is constrained within a flexible, hydrophilic-coated, 15-Fr outer diameter catheter.

This device is unique, as relative to the proximal stent graft edge, the midpoint of the proximal sealing ring is 7 mm distal with the Ovation Alto, as opposed to 13 mm distal with the Ovation iX.^1^ Furthermore, rings are located within each leg. The rings are filled with a low-viscosity, radiopaque liquid polymer using an auto-injector device after the deployment of a suprarenal nitinol stent with anchors. However, the route of the injection may potentially become blocked due to stent graft twisting, resulting in insufficient polymer filling within the rings. Furthermore, since the stent is radiolucent, visualizing the twisting is difficult until the polymer is injected; it can only be confirmed after polymer injection. The position of the contralateral gate cannot be visualized until the polymer is filled, making cannulation from the contralateral side challenging. Additionally, the passage of the wire through the crossover lumen may be difficult due to occlusion. Cannulation using a steerable catheter from the same side or cannulation from above the renal artery can be considered options for bail-out maneuvers. The mid-term outcomes of this Ovation Alto device have been published^2^^,^^3^; however, no reports of intraoperative leg occlusion of the stent graft’s main body exist.

We report a case of a patient diagnosed with an abdominal aortic aneurysm (AAA) who was surgically treated using EVAR with an Ovation Alto stent graft. Polymer underfilling due to contralateral leg twisting of the stent graft’s main body resulted in limb occlusion. The study protocol was reviewed and approved by Sendai Kosei Hospital Institutional Review Board (IRB approval no: 5-38; date: December 28, 2023). Informed consent was obtained from the patient for publication of this case report.

## Case Report

**Fig 1:**
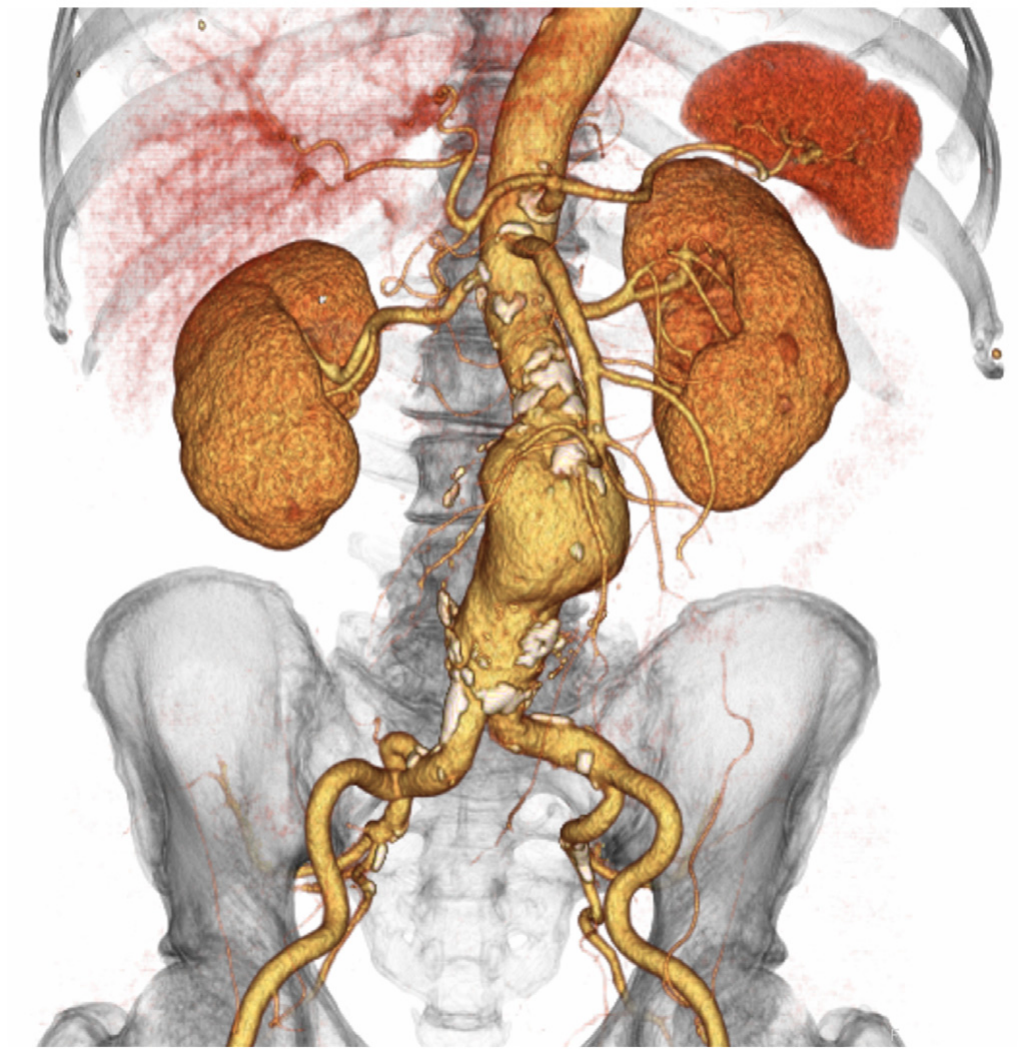
Preoperative three-dimensional reconstruction of a contrast-enhanced computed tomographic (*CECT*) image is depicted. CECT reveals an abdominal aortic aneurysm of 63 mm in a 70-year-old male patient.

**Fig 2:**
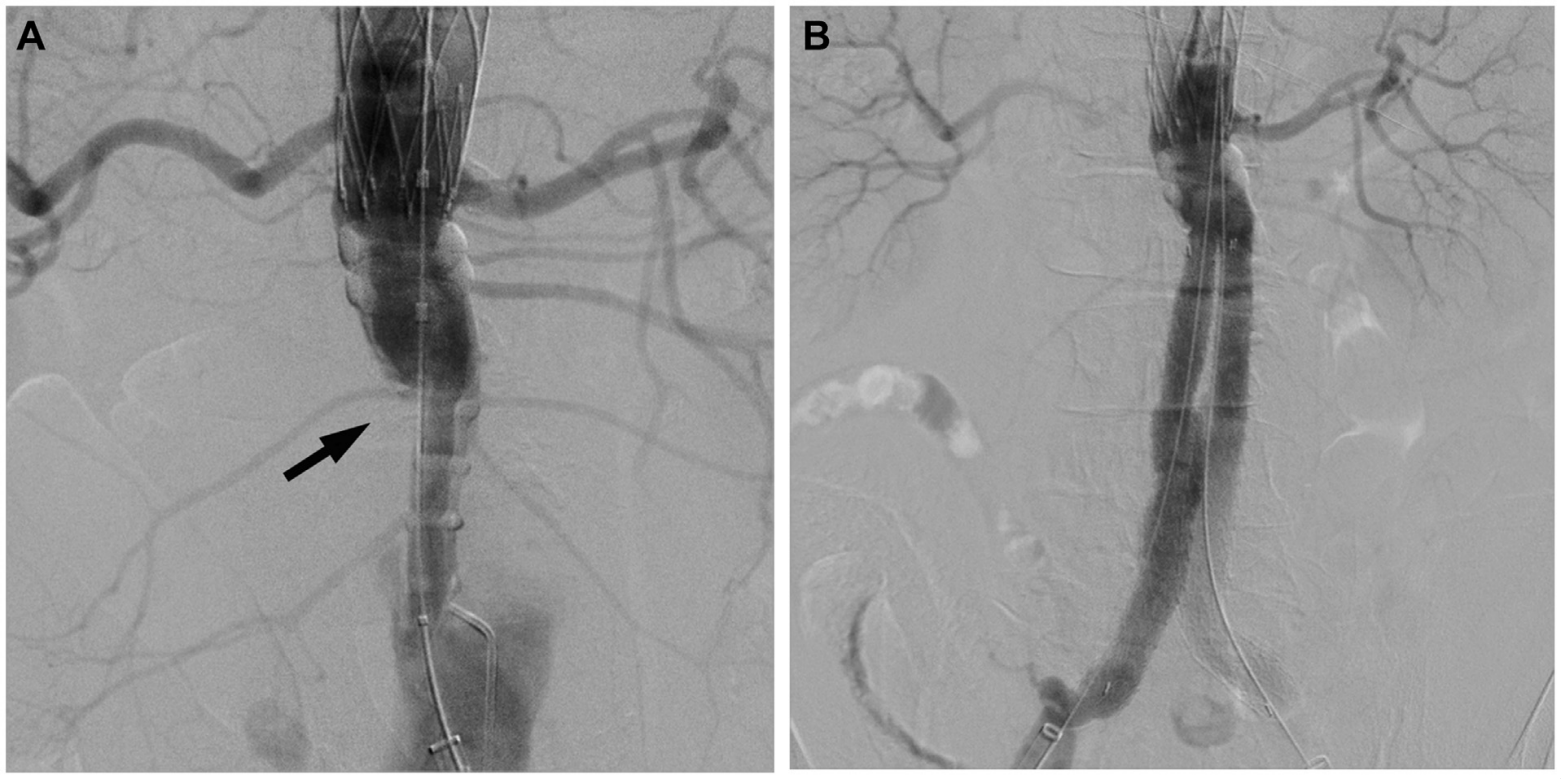
Intraoperative angiographic findings are shown. **A,** Suprarenal angiographic image subsequent to polymer injection is depicted. The right leg has not been filled with polymer and has been occluded from the level of the bifurcation of the main body (as indicated by the *black arrow*). **B,** A final angiographic image reveals that there is neither a stenosis nor an endoleak of the stent graft.

A 70-year-old male patient was diagnosed with an AAA of 63 mm by contrast-enhanced computed tomography (Fig 1) and was scheduled to undergo surgical treatment using EVAR with an Ovation Alto stent graft. The Alto device was selected because we believed that landing with the O-ring at a relatively favorable site between the left renal artery bifurcation and the calcification in the neck, where the characteristics are relatively better, would be beneficial. Informed consent for performing this procedure was obtained from the patient preoperatively. Under general anesthesia, the Perclose Prostyle device (Abbott Vascular), an 8-Fr catheter system, was used for the percutaneous delivery of surgical sutures to the bilateral femoral arteries to close the sheath entry sites at the end of the puncture. A 16-Fr sheath (DrySeal; WL Gore & Associates) was advanced over a super stiff wire, the Amplatz Super Stiff guidewire (Boston Scientific). Before the main body of the stent graft was delivered through the DrySeal introducer that was previously positioned in the aorta, we adjusted the anatomical position of the radiopaque markers under fluoroscopic guidance to ensure that the contralateral gate was oriented parallel to the common iliac artery. The main body of the stent graft (Alto Abdominal Stent Graft System TV-AB2680-N, Endologix Inc) was advanced to the infrarenal abdominal aorta through the Dryseal introducer. After the main body of the stent graft was inserted in the suprarenal position, the contralateral gate and stent graft’s main body within the device sheath were slightly rotated for finer adjustments. After the deployment of the uncovered proximal stent, the polymer was injected using an injection device. However, the polymer filling was insufficient, only filling the neck and ipsilateral leg portions while leaving the contralateral leg portion underfilled (Fig 2, *A*). Suprarenal angiography revealed that the contralateral leg was occluded. We could not detect the contralateral gate because the leg was radiolucent, with rings not filled with polymer. We inserted a 0.018″ guidewire (Command; Abbott) to crossover the lumen; however, we could not advance the guidewire owing to an obstruction in the middle of the ipsilateral leg. We also inserted a 0.014 guidewire (Command; Abbott); however, we could not advance. Cannulation of the infrarenal main body was performed using a 6-Fr destination catheter from the left brachial artery percutaneously. Cannulation of the contralateral leg with a hydrophilic soft-type guidewire (Radifocus; Terumo) was achieved using a 5-Fr impress catheter (Merit Medical). An EnSnare device (Atrieve Vascular Snare; Angiotech) was inserted from the right 8-Fr sheath; the hydrophilic soft-type guidewire was caught using the pull-through technique. After balloon dilatation of the contralateral leg of the main body using an 8-mm Mustang balloon dilatation catheter (Boston Scientific), the right leg of the Ovation iX (TV-IL1416140-J; Endologix Inc) was advanced and deployed from the level of the bifurcation of the main body to the right common iliac artery. Final angiography revealed that there was neither an endoleak nor a stenosis of the stent graft (Fig 2, *B*). The postoperative course was uneventful, and the post-procedural contrast-enhanced computed tomography confirmed the absence of an endoleak and stenosis of the stent graft (Fig 2).

## Discussion

We report a case in which a 70-year-old patient diagnosed with an AAA was surgically treated using an Ovation Alto stent graft, resulting in leg occlusion of the stent graft’s main body. As options for bail-out, maneuvers by endovascular cannulation using a steerable catheter from the same side or cannulation from above the renal artery can be considered. If endovascular treatment is difficult, open conversion may be required; however, in this case, the situation was managed endovascularly. This report highlights the importance of avoiding torsion of this device to circumvent potential twisting of the stent graft within the sheath after the insertion of the stent graft’s main body.

**Fig 3:**
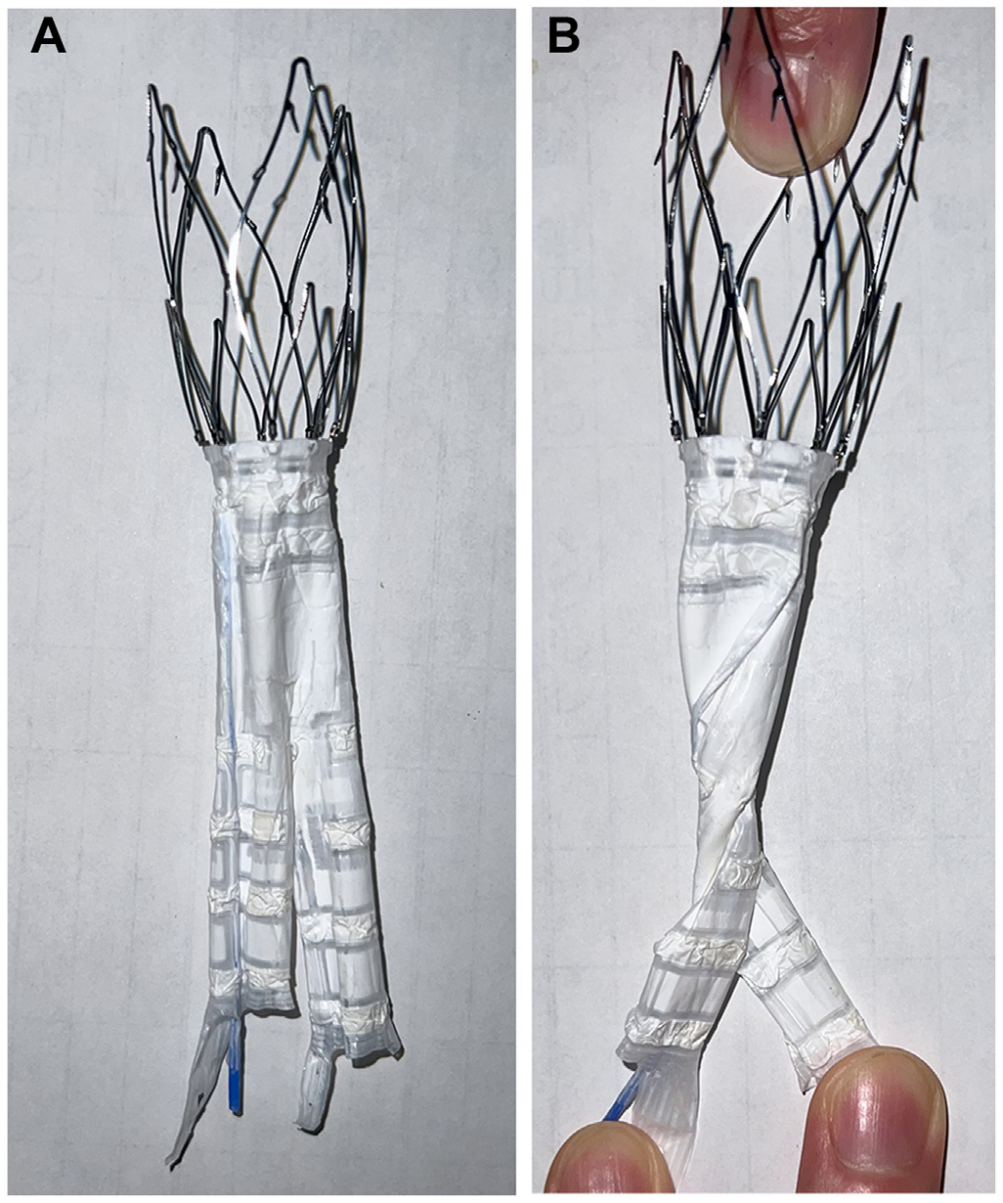
The Ovation Alto stent graft main body is depicted in vitro. **A,** The stent graft main body prior to polymer injection is depicted. **B,** Depiction of the stent graft main body lacking a radial force for self-expansion; thus, the limbs are susceptible to occlusion, due to stent graft twisting.

This stent graft lacks self-expanding capability (Fig 3); therefore, when the inner tube rotates, the leg of the stent graft’s main body within the sheath may rotate and bend. This may result in the leg remaining bent on deployment, thus causing it to be fixed in the aorta, with resultant occlusion.

In our case, after the stent graft’s main body was inserted in the suprarenal position, the contralateral gate and stent graft’s main body within the sheath were slightly rotated. Moreover, we rotated the outer sheath in addition to the inner tube for the adjustment of the radiopaque markers, with the potential for the stent graft to bend inadvertently. Furthermore, we considered that if the contralateral limb overlaps with the neck due to a long neck and the neck is narrow, rotating the graft in that narrow space can easily cause twisting.

The Ovation stent graft has demonstrated excellent short- and long-term outcomes in 5-year follow-ups.^4^, ^5^, ^6^ In these studies, adverse events did not occur due to inadequate polymer filling with secondary leg occlusion of the stent graft’s main body intraoperatively. Alsheekh et al reported a case of a symptomatic aortic body graft stenosis in the unsupported segment below the O-rings and hypothesized that the unique design of this stent graft may be subjected to external compressive forces or twisting of the stent graft’s main body.^7^ In our case, the diameter of the abdominal aorta below the O-ring was narrowed; nevertheless, considerable calcification or plaque that may have caused external compression was absent. Thus, we hypothesized that the probable cause was leg occlusion due to the leg twisting of the stent graft’s main body. Visually confirming the status of the graft at each angiographic step is crucial to avoid or recognize such graft twisting.

## Conclusion

We definitively report a rare case of leg occlusion of the main body of the Ovation Alto stent graft. This report highlights the importance of avoiding the rotation of this device to circumvent the potential twisting of the stent graft within the sheath after the insertion of the stent graft’s main body. In cases with a long neck and a relatively small diameter, there is a risk of twisting with the Alto device. Therefore, other devices may be considered.

## Disclosures

None.
